# The rs1550117 A>G variant in DNMT3A gene promoter significantly increases non-small cell lung cancer susceptibility in a Han Chinese population

**DOI:** 10.18632/oncotarget.15625

**Published:** 2017-02-22

**Authors:** Jingdong Wang, Chade Li, Fengting Wan, Zhou Li, Jingli Zhang, Jiankun Zhang, Xianhong Feng, Liang Tang, Bifeng Chen

**Affiliations:** ^1^ Department of Biological Science and Technology, School of Chemistry, Chemical Engineering and Life Sciences, Wuhan University of Technology, Wuhan, China; ^2^ Clinical Laboratory, Wuhan Xinzhou District People's Hospital, Wuhan, China; ^3^ Department of Human Anatomy, Histology and Embryology, Institute of Neuroscience, Changsha Medical University, Changsha, China

**Keywords:** non-small cell lung cancer, DNMT3A, rs1550117, Han Chinese population

## Abstract

In this study, we conducted a case-control study to explore the association between rs1550117 A>G variant of DNMT3A gene promoter and non-small cell lung cancer (NSCLC) susceptibility in a Han Chinese population. The genotyping of rs1550117 A>G variant was performed by polymerase chain reaction-restriction fragment length polymorphism (PCR-RFLP) and confirmed by sequencing. Allele G of rs1550117 was associated with an increased risk of NSCLC. Moreover, individuals carrying the GG genotypes had a higher risk to develop NSCLC than the AA and GA genotype carriers. Further stratified analysis showed that rs1550117 A>G was significantly related to age (> 60 years), male, smoking and drinking. *In vivo* detection of DNMT3A mRNA levels in NSCLC tissues and *in vitro* luciferase assays consistently showed that the allele G significantly decreased DNMT3A transcription. Additional functional analysis revealed that the increased binding affinity of transcription repressor SP1, which was associated with allele G of rs1550117, led to the significant decreased expression of DNMT3A. Collectively, our results propose a suppression role of DNMT3A in NSCLC development and emphasize the dual roles of DNMT3A in tumorigenesis.

## INTRODUCTION

Lung cancer has been the leading cancer diagnosed and cause of cancer death for many years in China, especially in Hubei province with a high incidence and mortality rate [1, 2]. Non-small cell lung cancer (NSCLC) is the most common type of lung cancer, accounting for 80%–90% [3]. Nowadays, surgical resection has been an efficient curative treatment for lung cancer patients in earlier stage [2]. Unfortunately, most lung patients are diagnosed in stage IIIB/IV, during which period the tumor are unresectable anymore. In view of this, the discovery of novelty strategy for risk prediction and early diagnosis of lung cancer is urgently needed.

DNA methylation is one of the best elucidated epigenetic modifications and has an important role in cancer development [4]. Of note, aberrant DNA methylation patterns have been found in most human cancers, including NSCLC [5]. As a *de novo* DNA methyltransferase, DNMT3A contributes to the establishment of genomic DNA methylation patterns [6], indicating that abnormal DNMT3A expression may be responsible for the aberrant DNA methylation in carcinogenesis. On the other side, certain genetic variants in the 5′- and 3′-UTR (untranslated region) of genes were recently proved to influence promoter activity (gene expression) and messenger RNA (mRNA) conformation (stability) [7]. Therefore, identification of functional variants in DNMT3A gene and analysis of their effects may lead to a better understanding of their impact on DNMT3A gene expression and individual susceptibility to cancer.

Recently, a number of studies have investigated the association between DNMT3A variants and cancer risk [8–15], and proposed a putative functional variant (rs1550117) in the 448bp upstream of the transcription start site of DNMT3A gene promoter [10]. However, the results from previous studies remain conflicting rather than conclusive [16, 17]. This discrepancy may be largely attributed to the insufficient sample sizes and different ethnic populations. Moreover, to the best of our knowledge, the association of DNMT3A rs1550117 with NSCLC susceptibility was still not elucidated. To address these issues, a case-control study was conducted to estimate the association between DNMT3A rs1550117 A>G variant and NSCLC risk in Hubei Han Chinese population with larger sample size.

## RESULTS

### Characteristics of study subjects

The distributions of age, gender, smoking status and alcohol status did not differ significantly between NSCLC patients and normal controls, suggesting that matching based on these four variables was adequate (Table 1). Moreover, the NSCLC patients and normal controls had a similar distribution of mean age: 60.1 years (range: 23~81 years) and 58.6 years (range: 27~85 years), respectively.

**Table 1:** Characteristics of the studied population of NSCLC patients and normal controls

Variables		NSCLC patients (*n* = 600)	Normal controls (*n* = 998)	*p* value^2^
Age (years)	≤ 60	330 (55.0%)^1^	556 (55.7%)	0.782
	> 60	270 (45.0%)	442 (44.3%)	
Gender	Male	428 (71.3%)	696 (69.7%)	0.499
	Female	172 (28.7%)	302 (30.3%)	
Smoking status	Ever	167 (27.8%)	250 (25.1%)	0.220
	Never	433 (72.2%)	748 (74.9%)	
Alcohol status	Ever	192 (32.0%)	294 (29.5%)	0.285
	Never	408 (68.0%)	704 (70.5%)	

^1^ Numbers in parentheses, percentage.

^2^ Age, gender, smoking status and alcohol status distributions of NSCLC patients and normal controls were compared using two-sided χ^2^ test.

### The DNMT3A rs1550117 A>G variant significantly increases the risk of NSCLC

The genotype frequencies of rs1550117 A>G variant were in agreement with Hardy-Weinberg equilibrium (HWE) in normal controls (*p* = 0.537), suggesting the enrolled control subjects were representative. In Table 2, it was presented that the genotype distributions of rs1550117 were significantly different between the NSCLC patients and normal controls (*p* = 0.001). Moreover, the G allele frequency was significantly higher among NSCLC patients than normal controls (*p* = 0.001, OR = 1.36, 95%CI = 1.18–1.71), indicating allele G was associated with an increased risk of NSCLC. Similarly, we also found a significant association between GG genotype of rs1550117 A>G variant and increased risk of NSCLC in three genetic models: GG *vs*. GA (*p* = 0.010, OR = 1.33, 95%CI = 1.06–1.71), GG *vs*. AA (*p* = 0.032, OR = 1.95, 95%CI = 1.03–3.60) and GG *vs*. GA+AA (*p* = 0.002, OR = 1.39, 95%CI = 1.15–1.80). These results indicated that the DNMT3A 5′-regulatory variant rs1550117 A>G significantly increases the risk of NSCLC. In addition, there were no significant different frequencies of DNMT3A rs1550117 in NSCLC patients at age range ≤ 60 years *vs*. > 60 years (*p*_genotype_ = 0.768, *p*_allele_ = 0.603), male *vs*. female (*p*_genotype_ = 0.656, *p*_allele_ = 0.607), smoking *vs*. non-smoking (*p*_genotype_ = 0.347, *p*_allele_ = 0.224), and drinking *vs*. non-drinking (*p*_genotype_ = 0.482, *p*_allele_ = 0.811) (Table 3).

**Table 2:** Genotype and allele distributions of DNMT3A rs1550117 A>G variant in NSCLC patients and normal controls, and their association with the risk of NSCLC

rs1550117 A>Gvariant	NSCLCpatients	Normalcontrols	*p*^2^	Logistic Regression	
				Genetic comparison	*p*^2^, OR(95% CI)^3^
G	1027(85.6%)^1^	1619(81.1%)	0.001	G *vs*. A	0.001, 1.36(1.18–1.71)
A	173(14.4%)	377(18.9%)			
GG	441(73.5%)	662(66.3%)	0.001	GG *vs*. GA	0.010, 1.33(1.06–1.71)
GA	145(24.2%)	295(29.6%)		GG *vs*. AA	0.032, 1.95(1.03–3.60)
AA	14(2.3%)	41(4.1%)		GA *vs*. AA	0.264, 1.45(0.77–2.75)
				GG *vs*. GA+AA	0.002, 1.39(1.15–1.80)
				GG+GA *vs*. AA	0.058, 1.80(1.00–3.35)

^1^ Numbers in parentheses, percentage.

^2^ The *p* value was calculated using two-sided χ^2^ test.

^3^ Adjusted for age, gender smoking status and alcohol status.

**Table 3:** The genotypes and allele frequencies of DNMT3A rs1550117 A>G in NSCLC patients

Variables		Genotype			*p* value^1^	Allele		*p* value^2^
		GG	GA	AA		G	A	
Total		441	145	14		1027	173	
Age	≤ 60	246	76	8	0.768	568	92	0.603
	> 60	195	69	6		459	81	
Gender	Male	312	107	9	0.656	731	125	0.607
	Female	129	38	5		296	48	
Smoking status	Ever	130	33	4	0.347	293	41	0.224
	Never	313	110	10		736	130	
Alcohol status	Ever	144	42	6	0.482	330	54	0.811
	Never	297	103	8		697	119	

^1^ Two-sided χ^2^ test for genotype distribution.

^2^ Two-sided χ^2^ test for allele distribution.

### The susceptibility to NSCLC with rs1550117 A>G variant is strongly related with age (> 60 years), male, smoking and drinking

Age, sex, smoking and drinking have been regarded as important factors in lung carcinogenesis [1, 2]. In this study, a stratified analysis of rs1550117 according to age, sex, smoking status and alcohol status was performed. The results showed that these factors would affect the association between rs1550117 A>G variant and NSCLC risk. Specifically, the individuals carrying G allele and GG genotype exhibited significantly increased NSCLC risk compared with individuals carrying A allele and GA/AA genotypes in > 60 years, male, smoking and drinking subgroups, while not in ≤ 60 years, female, non-smoking and non-drinking subgroups (Table 4). Moreover, all genotype frequencies were in agreement with the HWE among normal controls in each subgroup (*p* > 0.05). These results suggested that the rs1550117 A>G variant confers an increased risk to NSCLC, particularly in over than 60 years old males who smoke and drink.

**Table 4:** Stratification analysis of DNMT3A rs1550117 genotype and allele according to age, gender, smoking status and drinking status in NSCLC patients

Groups GG		Genotype			Allele		Logistic Regression [*p*^1^, OR(95% CI)]					
		GG	GA	AA	G	A	G *vs*. A	GG *vs*. GA	GG *vs*. AA	GA *vs*. AA	GG *vs*. GA+AA	GG+GA *vs*. AA
≤ 60 y	NSCLC patients	246	76	8	568	92	0.119, 1.24 (0.95–1.63)	0.062, 1.35 (0.99–1.86)	0.925, 1.04 (0.43–2.56)	0.582, 0.77 (0.31–1.94)	0.073, 1.32 (0.97–1.80)	0.935, 0.96 (0.40–2.35)
	Normal controls	383	160	13	926	186						
> 60 y	NSCLC patients	195	69	6	459	81	0.002, 1.56 (1.17–2.08)	0.074, 1.37 (0.97–1.93)	0.010, 3.26 (1.33–8.03)	0.066, 2.39 (0.94–6.03)	0.013, 1.52 (1.09–2.11)	0.017, 2.98 (1.22–7.28)
	Normal controls	279	135	28	693	191						
Male	NSCLC patients	312	107	9	731	125	0.003, 1.42 (1.13–1.79)	0.014, 1.41 (1.07–1.85)	0.062, 2.08 (0.96–4.47)	0.336, 1.47 (0.67–3.24)	0.005, 1.46 (1.12–1.90)	0.106, 1.88 (0.86–4.04)
	Normal controls	451	218	27	1120	272						
Female	NSCLC patients	129	38	5	296	48	0.168, 1.30 (0.90–1.88)	0.347, 1.24 (0.79–1.94)	0.313, 1.71 (0.60–4.86)	0.562, 1.38 (0.46–4.12)	0.233, 1.29 (0.85–1.98)	0.360, 1.62 (0.58–4.59)
	Normal controls	211	77	14	499	105						
Smoking	NSCLC patients	130	33	4	293	41	0.0003, 2.06 (1.40–3.04)	0.0002, 2.38 (1.50–3.78)	0.142, 2.40 (0.75–7.72)	0.989, 1.00 (0.30–3.39)	0.0001, 2.38 (1.53–3.71)	0.289, 1.88 (0.59–5.99)
	Normal controls	149	90	11	388	112						
Non- smoking	NSCLC patients	313	110	10	736	130	0.090, 1.22 (0.97–1.53)	0.353, 1.14 (0.87–1.49)	0.104, 1.83 (0.88–3.80)	0.215, 1.61 (0.76–3.42)	0.181, 1.20 (0.92–1.55)	0.124, 1.77 (0.86–3.65)
	Normal controls	513	205	30	1231	265						
Drinking	NSCLC patients	144	42	6	330	54	0.001, 1.82 (1.29–2.57)	0.008, 1.77 (1.16–2.71)	0.031, 2.80 (1.10–7.12)	0.359, 1.58 (0.60–4.20)	0.002, 1.90 (1.27–2.84)	0.066, 2.39 (0.94–6.02)
	Normal controls	180	93	21	453	135						
Non- drinking	NSCLC patients	297	103	8	697	119	0.109, 1.22 (0.96–1.54)	0.182, 1.21 (0.92–1.60)	0.309, 1.54 (0.67–3.54)	0.577, 1.28 (0.54–2.99)	0.129, 1.23 (0.94–1.61)	0.369, 1.46 (0.64–3.35)
	Normal controls	482	202	20	1166	242						

^1^The p value was calculated using two-sided χ^2^ test.

### The rs1550117 A>G variant decreases DNMT3A transcriptional activity in NSCLC

**Figure 1:**
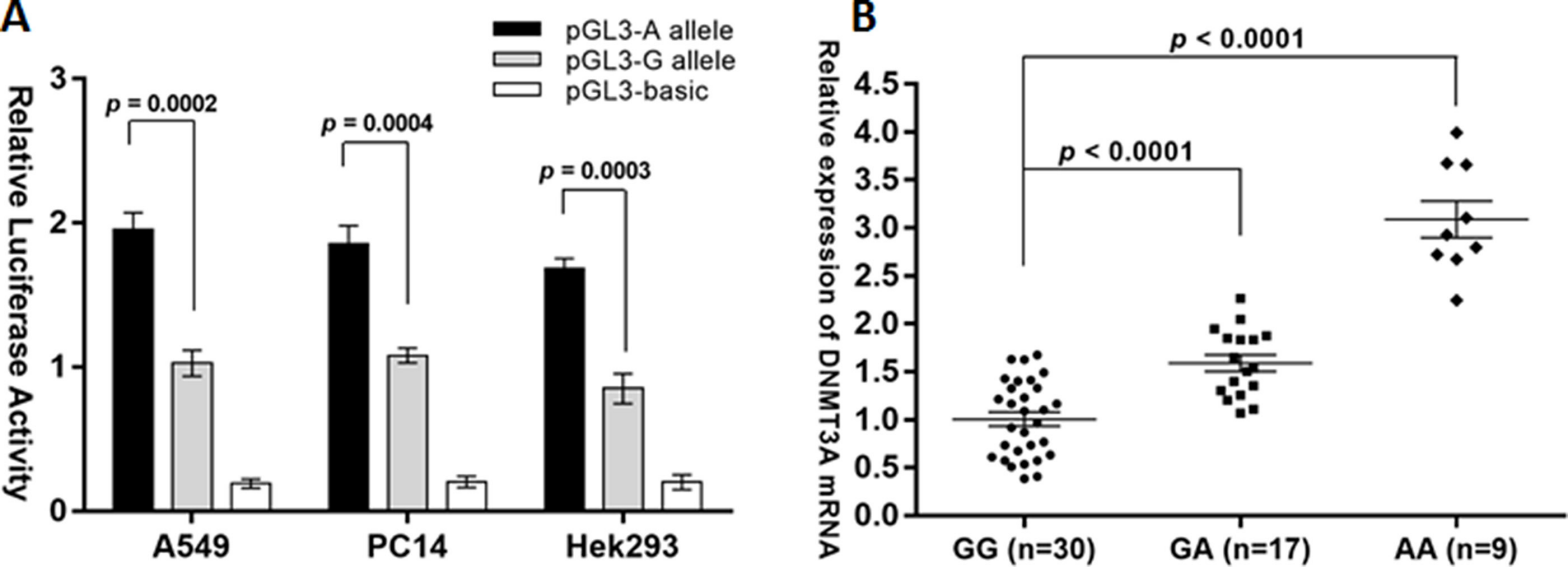
The DNMT3A rs1550117 A>G variant decreases DNMT3A expression at the transcriptional level (**A**) Luciferase activity was significantly increased in the A allelic construct compared with the G construct in two NSCLC cells (A549 and PC14) and Hek293 cells. (**B**) Quantitative real-time RT-PCR analysis of DNMT3A *in vivo* mRNA levels in 56 NSCLC tissue samples with different genotypes.

Although previous study demonstrated that the rs1550117 A>G variant affected the transcriptional activity of DNMT3A promoter in Chinese hamster ovary cells [10], it was wondered that whether this underlying mechanism was also applicable in NSCLC. We performed dual luciferase assays to find that the plasmid containing the G allele showed a significantly lower luciferase activity than the A allele with a 48% decrease in A549 cells, a 45% decrease in PC14 cells and a 50% decrease in Hek293 cells (Figure 1A). It was recently identified a novel short isoform-DNMT3A2 protein (about 82 kDa). Transcription of this isoform is initiated from a different promoter in the sixth intron of the DNMT3A gene, which encodes the full length isoform-DNMT3A protein (about 120 kDa). Therefore, the rs1550117 A>G variant in DNMT3A gene promoter may be specifically responsible for the expression of DNMT3A but not DNMT3A2. In this study, the DNMT3A mRNA levels in 56 NSCLC tissue samples with different rs1550117 A>G genotypes were also tested, and DNMT3A was significantly upregulated in GA samples (1.6-fold) and AA samples (3.1-fold) than in GG samples (Figure 1B). These results consistently suggested that the rs1550117 A>G variant decreases DNMT3A transcriptional activity in NSCLC.

### The rs1550117 A>G variant increases the transcription repressor SP1 binding affinity

**Figure 2:**
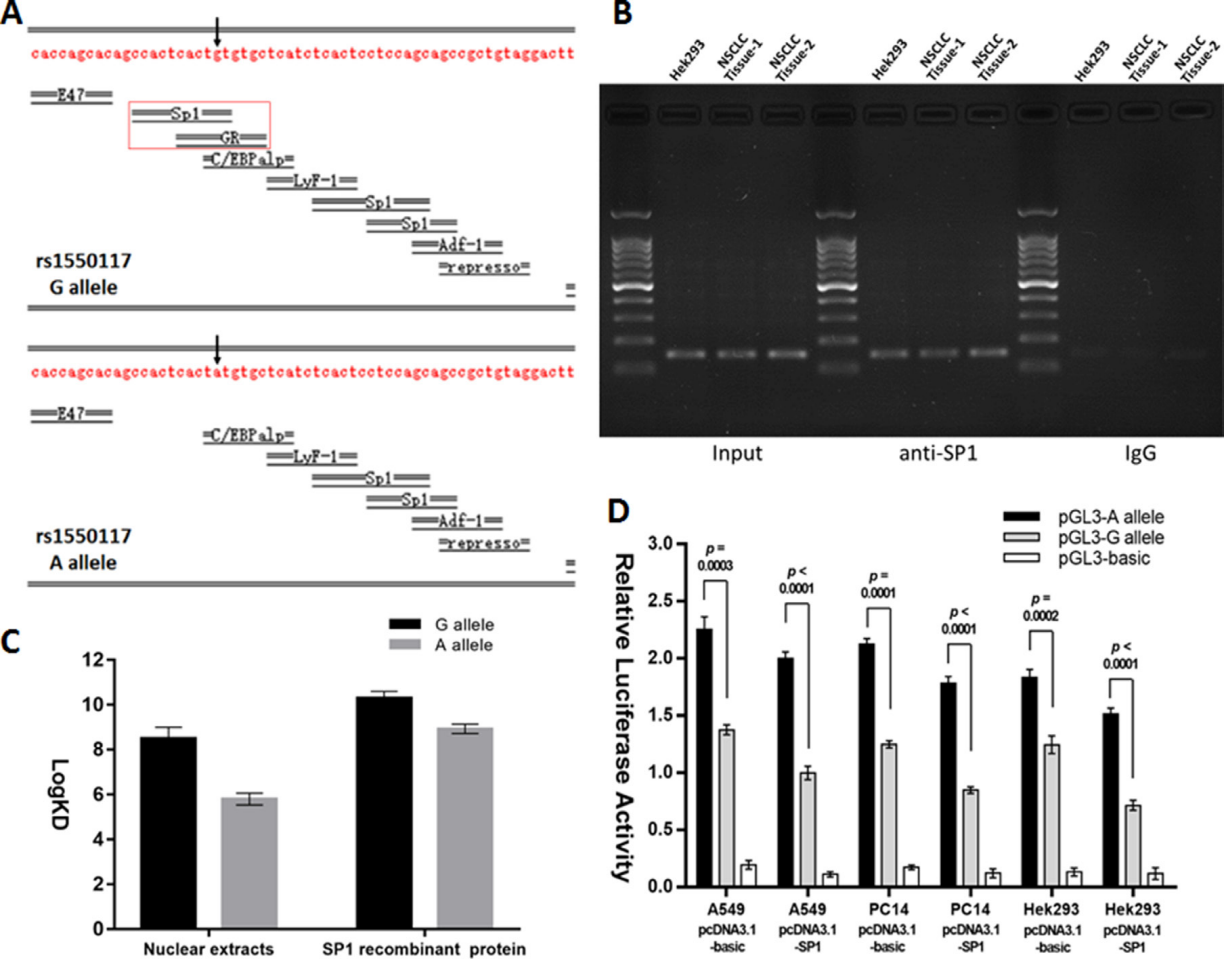
The rs1550117 A>G variant affects the transcription factor binding affinity (**A**) Computational analysis predicted transcription factors for DNMT3A rs1550117 A>G variant. (**B**) ChIP assays using Hek293 and two NSCLC tissue samples. The presence of the SP1-binding DNMT3A promoter was verified by PCR. (**C**) SPR analysis comparing the binding affinity of Hek293 nuclear extracts or SP1 recombinant protein to DNA probes containing either the rs1550117 A or G alleles. (**D**) A luciferase construct containing either the A or G allele was co-transfected with pcDNA3.1-basic (control) or pcDNA3.1-SP1 expression plasmids in A549, PC14 and Hek293 cells.

Alibaba2 software (http://gene-regulation.com/pub/programs/alibaba2/index.html?) was used to predicted that the rs1550117 A>G variant creates the transcription factor (TF) binding sites for SP1 and GR (Figure 2A). However, the chromatin immunoprecipitation (ChIP) sequencing results in the ChIPBase v2.0 database (http://rna.sysu.edu.cn/chipbase/) and previous investigation results collectively suggested that SP1 but not GR could bind to the DNMT3A promoter region [18, 19]. In this study, through ChIP assays, it was demonstrated that the DNMT3A promoter fragment with −448 site was occupied by SP1 (Figure 2B). Moreover, the surface plasma resonance (SPR) analysis revealed that, compared with the A allele oligonucleotide probe, the G allele oligonucleotide probe had higher binding affinity to Hek293 nuclear proteins or purified recombinant SP1 protein (Figure 2C). The co-transfection experiment showed that the ectopic SP1 expression generally decreased the luciferase activities of the plasmids containing DNMT3A rs1550117 A allele or G allele, and the rs1550117 variant amplified the promoter function disparity (Figure 2D). Taken together, SP1 acts as a transcription repressor of DNMT3A gene, and the rs1550117 A>G increases the binding affinity of SP1 to the DNMT3A promoter, which finally contributes to the decreased expression of DNMT3A.

## DISCUSSION

DNMT3A was previously suggested to promote tumorigenesis [20]. However, the underlying molecular mechanism remains elusive. One possibility is that overexpressed DNMT3A may lead to the silencing of certain tumor suppressor genes (TSGs) in tumorigenesis. Indeed, it was showed that knockdown of DNMT3A would upregulate the expression of some immune response genes in melanoma [21]. Similarly, depletion of DNMT3A restored the expression of various TSGs (including PTEN) that participate in cell cycle regulation, transcription regulation, and signal transduction in hepatocellular carcinoma (HCC) [22].

On the other side, numerous genetic association studies had investigated the association between DNMT3A rs1550117 A>G variant and cancer risk in gastric cancer, urothelial cancer, esophageal cancer, ovarian cancer, breast cancer, colorectal cancer and hepatocellular cancer, but the results were inconsistent [8–15]. In view of this, Zhang et al. conducted a comprehensive meta-analysis and found that the rs1550117 A allele significantly increased the cancer risk [16]. The interesting question is why the rs1550117 variant could affect individual susceptibility to cancer. In this study, it was firstly revealed that SP1 acts as a transcription repressor of DNMT3A gene, and SP1 possesses a higher binding affinity to rs1550117 G allele than to A allele. Therefore, compared with the G allele, the rs1550117 A allele would decrease SP1 binding affinity, and then leads to the increased expression of DNMT3A, which finally increases cancer susceptibility.

Although these evidences clearly assigned an oncogenic role to DNMT3A in tumorigenesis, the recently discovered DNMT3A mutations in acute myeloid leukemia (AML) suggested that its role in cancer is more complex than previously thought [23–27]. Further functional analysis illustrated that the DNMT3A mutations would affect global DNA methylation pattern and expression of HOXA genes [28], which act an important role in AML tumorigenesis [29]. In addition, two latest publications showed that Dnmt3a deletion in a mouse lung cancer model promoted tumor progression but not initiation [30, 31], and Dnmt3a maintained the methylation-dependent repression (MDR) of specific oncogenes which were involved in key steps of lung tumor progression, including angiogenesis, cell adhesion and movement [30]. These results suggested that Dnmt3a may be a tumor suppressor gene and a critical determinant of lung tumorigenesis [30]. However, it leaves a question that whether DNMT3A acts the same in human lung cancer as in mouse lung cancer model.

In this study, the putative functional rs1550117 A>G variant in DNMT3A gene promoter was selected to evaluate the association between DNMT3A gene and NSCLC susceptibility in a Han Chinese population. Interestingly, we found that individuals carrying rs1550117 GG genotype exhibited significantly increased NSCLC susceptibility compared with individuals carrying GA and AA genotypes, suggesting that G allele was a harmful effect potentially exhibited by rs1550117 variant. Meanwhile, the GG genotype was associated with lower DNMT3A mRNA expression in NSCLC tissues than GA and AA genotypes. Therefore, DNMT3A may participate in the suppression of NSCLC in human, which was consistent with the finding from previous study in mouse model with lung cancer.

The present findings firstly demonstrated that DNMT3A rs1550117 A>G variant is associated with a significantly increased risk of NSCLC in a Han Chinese population. Meanwhile, it was also firstly revealed that the DNMT3A rs1550117 A>G variant decreases DNMT3A expression by increased the binding affinity of transcriptional suppressor SP1. Moreover, the stratified analysis implied that over than 60 years old males who smoke and drink are more susceptible to NSCLC with rs1550117 A>G. Our study proposes an unintended role of DNMT3A in suppression of NSCLC development and further emphasizes the dual roles of DNMT3A in tumorigenesis. The results reported here may initiate a novel strategy for the prediction and prevention of NSCLC. However, further confirmatory studies should be undertaken in other ethnic populations because the present observations involved only Chinese Han population.

## MATERIALS AND METHODS

### Subjects

A total of 998 normal controls and 600 patients with histologically confirmed NSCLC were recruited in the current study. All subjects were Han Chinese living in Hubei province. Nowadays, more and more Chinese are inclined to have a physical examination every year. The normal controls were selected from cancer-free individuals who visited Wuhan Xinzhou District People's Hospital for annual physical examinations or who volunteered to participate in the epidemiology survey during the same period. It was required that the normal controls passed all annual physical examinations in the latest three years. The patients were confirmed histopathologically and volunteers recruited from the same hospital. This study was approved by the Ethical Committees of Wuhan Xinzhou District People's Hospital and Wuhan University of Technology, and written informed consent for the genetics analysis was obtained from all subjects or their guardians

### DNMT3A rs1550117 A>G variant genotyping

Samples were collected into blood vacuum tubes containing ethylenediaminetetra-acetic acid (EDTA) and stored at 4°C. Genomic DNA was extracted within 1 week of sample collection by proteinase K digestion as previously described [32]. The transition of A>G of DNMT3A rs1550117 variant creates a TspRI restriction site, PCR-RFLP was used to detect this A-G transition in the promoter of DNMT3A at -448 A>G (GenBank accession No.NT_022184.14:g.4381840). The PCR reaction was performed in a total of 15μl containing 50ng genomic DNA, 1.5 μl 10× Taq Buffer (Mg^2+^ Plus), 0.2 μl 10 mM dNTP, 1 μl 1 mM Primer (forward: 5′-ACACACCGC CCTCACCCCTT-3′; reverse: 5′-TCCAGCAATCCC TGCCCACA-3′), and 0.5U Taq polymerase (Takara Biotechnology Co. Ltd, Dalian, China). PCR cycle conditions consisted of an initial melting step of 94°C for 5 min, followed by 36 cycles of 94°C for 30s, 63°C for 30 s, 72°C for 30 s and a final extension step of 72°C for 10 min. The 358bp fragment was then digested with TspRI (Takara Biotechnology Co. Ltd, Dalian, China) overnight at 37°C, the digested products were separated on a 2.0% agarose gel and the RFLP bands visualized under ultraviolet light with Gel-Red staining. The wild-type G allele consists of a TspRI restriction site that results in three bands (155 bp, 121 bp and 82 bp), while the A allele produces two bands (276 bp and 82 bp). For quality control, genotyping analysis was performed blind, with respect to case/control status, and repeated twice for all subjects. The results of genotyping were 100% concordant. In order to confirm the genotyping results, 20% randomly selected PCR-amplified DNA samples were examined by DNA sequencing, and the results were also 100% concordant.

### Plasmid constructs, host cell culture and dual luciferase assays

To construct the DNMT3A reporter plasmid, we amplified a 588bp DNMT3A promoter fragment from -684bp to -97bp by PCR from genomic DNA, which contains the A or G allele of rs1550117 A>G variant. It was notable that the amplified fragment contains the putative promoter sequence of DNMT3A gene (−312bp ~ -262bp: TCAGCACTTCAGCTATA TCACAGTGCCCTGAGCTCCCTGACTGGCACAGG), which was analyzed with BDGP online software (http://www.fruitfly.org/seq_tools/promoter.html). The PCR products were then subcloned into the NheI and HindIII restriction sites of the pGL3-Basic vector (Promega, Madison, WI, USA). We verified all of the recombinant clones by DNA sequencing. The primers utilized were: 5′-CTAGCTAGCTCAGCACTGGGGCTG-3′ (forward) and 5′-CCCAAGCTTCTGTGACGCTAAAA-3′ (reverse). 2 NSCLC cells (A549 and PC14) and the human embryonic kidney 293 (Hek293) cells (1 × 10^5^) were seeded in 24-well culture plates. After 24 h of culture, the host cells were co-transfected with the pGL3-Basic (blank control), pGL3-A allele or pGL3-G allele plasmids and the pRL-TK plasmid as a normalization control, half of the cells were additionally co-transfected with the pcDNA3.1-SP1 expression plasmid or equivalent amounts of pcDNA3.1-basic vector using Lipofectamine 2000 (Invitrogen, Carlsbad, CA, USA), according to the manufacturer's instructions. After an additional 24 h of culture, the transfected cells were assayed for luciferase activity using the Dual-Luciferase Reporter Assay System (Promega, Madison, WI, USA). Three independent transfection experiments were performed, and each luciferase assay was carried out in triplicate.

### Quantitative real-time RT-PCR

56 NSCLC tissue samples were obtained from NSCLC patients who had undergone surgical resection at the Wuhan Xinzhou District People's Hospital (Wuhan, Hubei Province, China). Total RNA was extracted from the human NSCLC samples preserved in RNAlater (Qiagen, Valencia, CA, USA) and converted to cDNA using random hexamers, oligo (dT) primers and Moloney murine leukemia virus reverse transcriptase (Takara Biotechnology Co. Ltd, Dalian, China). The DNMT3A mRNA levels were measured by quantitative real-time RT-PCR using the Applied Biosystems 7900HT Fast Real-Time PCR System (Applied Biosystems, Foster City, CA, USA), and GAPDH was used as an internal reference gene. Each reaction was performed in triplicate. The primers used for DNMT3A amplification were 5′-ACCCAGCGCAGAAGCAG-3′ (forward) and 5′-A TAGATCCCGGTGTTGAGCC-3′ (reverse), the primers for GAPDH were 5′-TGCACCACCAACTGCTTAGC-3′ (forward) and 5′-GGCATGGACTGTGGTCATGAG-3′ (reverse). Relative quantification of DNMT3A mRNA was calculated by using the 2-ΔΔCT method, and each assay was done in triplicate.

### SPR analysis

The SPR analysis was carried out using the ProteOn XPR36 Protein Interaction Array System (Bio-Rad, Hercules, CA, USA). Biotinylated duplex oligonucleotide probes representing the rs1550117 A or G alleles (sequences were rs1550117 [A] Forward: 5′-CAGCCACTCACTATGTGCTCATCTC-3′, [A] Reverse: 5′-GAGATGAGCACATAGTGAGTGGCTG-3′; rs1550117 [G] Forward: 5′-CAGCCACTCACTGTGTGCTCATCTC -3′, [G] Reverse: 5′-GAGATGAGCACACAGTGAGTGG CTG-3′) were immobilized on the streptavidin-modified surfaces of the different channels from DNA solutions at a fixed concentration (400 nM) to ensure identical surface density. Nuclear extracts from Hek293 cells or purified SP1 recombinant protein were diluted in PBST (10 mM Na-phosphate, 150 mM NaCl and 0.005% Tween 20, pH 7.4) to different concentrations and then pre-incubated with non-specific DNA for 15 min before passing across the DNA immobilized surface. The results presented in the sensorgram were converted by BIA evaluation software. Each experiment was repeated three times.

### ChIP assays

The ChIP assays were performed using the EZ ChIP Kit (Upstate Lake Placid, NY, USA). First, Hek293 cells and two NSCLC tissue samples were crosslinked by 1% formaldehyde for 10 min. DNA was then sonicated into fragments with a mean length of 200 to 1000 bp. The sheared chromatin was immunoprecipitated by incubation with antibodies against SP1 or non-specific rabbit IgG (Santa Cruz Biotechnology, Santa Cruz, CA) overnight at 4°C. The DNA fragments were identified using PCR, and the primers utilized were: 5′- CACCGCCCTCACCCCATCA-3′ (forward) and 5′- TGCCCAGCCGCAAGTCCTA-3′ (reverse).

### Statistical analysis

The χ^2^ test was used to compare the difference in age, gender, smoking status and alcohol status between NSCLC patients and normal controls. Genotypic frequency of rs1550117 A>G variant was tested for departure from Hardy-Weinberg equilibrium (HWE) using the χ^2^ test. To evaluate the association between rs1550117 A>G variant and NSCLC risk, ORs and 95% confidence intervals (CIs) were calculated by unconditional logistic regression analysis with adjustments for age, sex smoking status and alcohol status. Other differences were evaluated using the Student's *t*-test. Data were expressed as means and standard deviations (SD) from at least three independent experiments. All statistical tests were two-tailed with *P* < 0.05 set as the significance level and were performed using SPSS 15.0 software (SPSS, Chicago, IL, USA).

## References

[1] Chen W, Zheng R, Zeng H, Zhang S (2015). Epidemiology of lung cancer in China. Thorac Cancer.

[2] Herbst RS, Heymach JV, Lippman SM (2008). Lung cancer. N Engl J Med.

[3] Siegel RL, Miller KD, Jemal A (2015). Cancer statistics, 2015. CA Cancer J Clin.

[4] Esteller M (2008). Epigenetics in cancer. N Engl J Med.

[5] Fernandez AF, Huidobro C, Fraga MF (2012). De novo DNA methyltransferases: oncogenes, tumor suppressors, or both?. Trends Genet: TIG.

[6] Lechner M, Boshoff C, Beck S (2010). Cancer epigenome. Adv Genet.

[7] Shastry BS (2009). SNPs: impact on gene function and phenotype. Methods Mol Biol.

[8] Cao XY, Jia ZF, Cao DH, Kong F, Jin MS, Suo J, Jiang J (2013). DNMT3a rs1550117 polymorphism association with increased risk of Helicobacter pylori infection. Asian Pac J Cancer Prev.

[9] Chung CJ, Chang CH, Liu CS, Huang CP, Chang YH, Chien SN, Tsai PH, Hsieh HA (2014). Association of DNA methyltransferases 3A and 3B polymorphisms, and plasma folate levels with the risk of urothelial carcinoma. PloS one.

[10] Fan H, Liu D, Qiu X, Qiao F, Wu Q, Su X, Zhang F, Song Y, Zhao Z, Xie W (2010). A functional polymorphism in the DNA methyltransferase-3A promoter modifies the susceptibility in gastric cancer but not in esophageal carcinoma. BMC Med.

[11] Mostowska A, Sajdak S, Pawlik P, Lianeri M, Jagodzinski PP (2013). DNMT1, DNMT3A and DNMT3B gene variants in relation to ovarian cancer risk in the Polish population. Mol Biol Rep.

[12] Sun MY, Yang XX, Xu WW, Yao GY, Pan HZ, Li M (2012). Association of DNMT1 and DNMT3B polymorphisms with breast cancer risk in Han Chinese women from South China. Genet Mol Res.

[13] Yang XX, He XQ, Li FX, Wu YS, Gao Y, Li M (2012). Risk-association of DNA methyltransferases polymorphisms with gastric cancer in the Southern Chinese population. Int J Mol Sci.

[14] Zhao C, Yan F, Wu H, Qiao F, Qiu X, Fan H (2013). DNMT3A -448A>G polymorphism and the risk for hepatocellular carcinoma. Biomed Rep.

[15] Zhao Z, Li C, Song Y, Wu Q, Qiao F, Fan H (2012). Association of the DNMT3A -448A>G polymorphism with genetic susceptibility to colorectal cancer. Oncology letters.

[16] Zhang W, Xu Y, Ma G, Qi W, Gu H, Jiang P (2015). Genetic Polymorphism of DNA Methyltransferase 3A rs1550117 A>G and Risk of Cancer: A Meta-analysis. J Invest Surg.

[17] Liu CH, Tao T, Jiang L, Xu B, Zhang L, Lu K, Zhang XW, Chen SQ, Liu DC, Chen M (2015). DNMT3A -448A>G polymorphism and cancer risk: a meta-analysis. Genet Mol Res.

[18] Jinawath A, Miyake S, Yanagisawa Y, Akiyama Y, Yuasa Y (2005). Transcriptional regulation of the human DNA methyltransferase 3A and 3B genes by Sp3 and Sp1 zinc finger proteins. Biochem J.

[19] Reddy TE, Pauli F, Sprouse RO, Neff NF, Newberry KM, Garabedian MJ, Myers RM (2009). Genomic determination of the glucocorticoid response reveals unexpected mechanisms of gene regulation. Genome Res.

[20] Esteller M (2007). Cancer epigenomics: DNA methylomes and histone-modification maps. Nat Rev Genet.

[21] Deng T, Kuang Y, Wang L, Li J, Wang Z, Fei J (2009). An essential role for DNA methyltransferase 3a in melanoma tumorigenesis. Biochem Biophys Res Commun.

[22] Zhao Z, Wu Q, Cheng J, Qiu X, Zhang J, Fan H (2010). Depletion of DNMT3A suppressed cell proliferation and restored PTEN in hepatocellular carcinoma cell. Biochem Biophys Res Commun.

[23] Metzeler KH, Walker A, Geyer S, Garzon R, Klisovic RB, Bloomfield CD, Blum W, Marcucci G (2012). DNMT3A mutations and response to the hypomethylating agent decitabine in acute myeloid leukemia. Leukemia.

[24] Ley TJ, Ding L, Walter MJ, McLellan MD, Lamprecht T, Larson DE, Kandoth C, Payton JE, Baty J, Welch J, Harris CC, Lichti CF, Townsend RR (2010). DNMT3A mutations in acute myeloid leukemia. N Engl J Med.

[25] Shah MY, Licht JD (2011). DNMT3A mutations in acute myeloid leukemia. Nat Genet.

[26] Hou HA, Kuo YY, Liu CY, Chou WC, Lee MC, Chen CY, Lin LI, Tseng MH, Huang CF, Chiang YC, Lee FY, Liu MC, Liu CW (2012). DNMT3A mutations in acute myeloid leukemia: stability during disease evolution and clinical implications. Blood.

[27] Fried I, Bodner C, Pichler MM, Lind K, Beham-Schmid C, Quehenberger F, Sperr WR, Linkesch W, Sill H, Wolfler A (2012). Frequency, onset and clinical impact of somatic DNMT3A mutations in therapy-related and secondary acute myeloid leukemia. Haematologica.

[28] Yan XJ, Xu J, Gu ZH, Pan CM, Lu G, Shen Y, Shi JY, Zhu YM, Tang L, Zhang XW, Liang WX, Mi JQ, Song HD (2011). Exome sequencing identifies somatic mutations of DNA methyltransferase gene DNMT3A in acute monocytic leukemia. Nat Genet.

[29] Eklund EA (2007). The role of HOX genes in malignant myeloid disease. Current opinion in hematology.

[30] Gao Q, Steine EJ, Barrasa MI, Hockemeyer D, Pawlak M, Fu D, Reddy S, Bell GW, Jaenisch R (2011). Deletion of the de novo DNA methyltransferase Dnmt3a promotes lung tumor progression. Proc Natl Acad Sci USA.

[31] Raddatz G, Gao Q, Bender S, Jaenisch R, Lyko F (2012). Dnmt3a protects active chromosome domains against cancer-associated hypomethylation. PLoS Genet.

[32] Miller SA, Dykes DD, Polesky HF (1988). A simple salting out procedure for extracting DNA from human nucleated cells. Nucleic Acids Res.

